# Supplementary material for: MMORF—FSL’s MultiMOdal Registration Framework
Source: Imaging Neurosci (Camb). Author manuscript; Available in PMC 2024 Dec 20. (PMC7617249; doi:10.1162/imag_a_00100)
Supplement: Supplementary file 1 [file EMS201863-supplement-Supplementary_Materials.pdf]

APPENDIX A. LEVENBERG-MARQUARDT AND MAJORISE-MINIMISATION OPTIMISATION

Appendix A.1. Levenberg-Marquardt

Given a quadratic Taylor approximation of a cost function $\mathcal{C}$ around the point $\mathbf{w}$ in parameter space,

$$\mathcal{C}(\mathbf{w}+\Delta\mathbf{w}) \approx \mathcal{C}(\mathbf{w}) + \nabla\mathcal{C}(\mathbf{w})^{\mathrm{T}}\Delta\mathbf{w} + \frac{1}{2}\Delta\mathbf{w}^{\mathrm{T}}\mathbf{H}(\mathbf{w})\Delta\mathbf{w} \quad \text{(A.1)}$$

where $\nabla\mathcal{C}$ and $\mathbf{H}$ are the gradient and the Hessian of the cost function, respectively. The update $\Delta\mathbf{w}$ that minimises this approximation to the cost function (when the cost function is convex) is then:

$$\Delta\mathbf{w} = -\mathbf{H}^{-1}(\mathbf{w})\nabla\mathcal{C}(\mathbf{w}) \quad \text{(A.2)}$$

For cost functions that can be written in the form:

$$\mathcal{C}(\mathbf{w}) = \frac{1}{2}\sum_{i=1}^{N} c_i^2(\mathbf{w}) \quad \text{(A.3)}$$

The gradient is then:

$$\nabla\mathcal{C}(\mathbf{w}) = \sum_{i=1}^{N} c_i(\mathbf{w})\frac{\partial c_i}{\partial \mathbf{w}} \quad \text{(A.4)}$$

And the Hessian is:

$$\mathbf{H}(\mathbf{w}) = \sum_{i=1}^{N} \frac{\partial c_i}{\partial \mathbf{w}}^{\mathrm{T}} \frac{\partial c_i}{\partial \mathbf{w}} + c_i(\mathbf{w})\frac{\partial^2 c_i}{\partial \mathbf{w}^2} \quad \text{(A.5)}$$

Gauss-Newton approximates $\mathbf{H}$ by dropping the second, mixed partial derivative, term in Equation A.5. Close to the optimum set of $\mathbf{w}$, the Newton and Gauss-Newton Hessians tend towards equivalence. Further from the optimum, the Gauss-Newton Hessian has the benefit of being positive definite, and therefore the approximation of the cost function is always convex. This ensures that each step is always in a direction that would improve the original cost function.

However, the Gauss-Newton step can still lead to an increase in the cost function if it oversteps. This can be addressed by the Levenberg-Marquardt (LM) extension (Nocedal & Wright, 2006, Ch.10, pp258), which replaces the Gauss-Newton Hessian ($\mathbf{H}_{GN}$) with:

$$\mathbf{H}_{LM} = \mathbf{H}_{GN} + \lambda\mathbf{I}. \quad \text{(A.6)}$$

For small values of λ, the update $\Delta\mathbf{w}$ behaves much like a Gauss-Newton update, and for large values of λ it behaves more like gradient descent. After choosing a sensible starting value for λ (e.g., 0.001), a typical Levenberg-Marquardt iteration then proceeds as follows:

- Calculate a candidate parameter update $\Delta\mathbf{w}$ using Equation A.2.
- If $\mathcal{C}(\mathbf{w}+\Delta\mathbf{w}) < \mathbf{C}(\mathbf{w})$ accept the update and set $\lambda = \lambda \div 10$.
- If $\mathcal{C}(\mathbf{w}+\Delta\mathbf{w}) > \mathbf{C}(\mathbf{w})$ set $\lambda = \lambda \times 10$ and recompute $\Delta\mathbf{w}$ and $\mathcal{C}(\mathbf{w}+\Delta\mathbf{w})$, repeating until $\mathcal{C}(\mathbf{w}+\Delta\mathbf{w}) < \mathcal{C}(\mathbf{w})$ and then accept the update.

Appendix A.2. Majorise-minimisation

The Majorise-Minimisation (MM) algorithm (Hunter & Lange, 2004) is a method for cost function minimisation that can be used when storing the full Gauss-Newton Hessian is infeasible (e.g., due to memory constraints, which prove to be important in this application). In essence, what this algorithm states is that if $z(\mathbf{w}) \geq y(\mathbf{w}), \forall\mathbf{w}$, with both y and z convex and touching, then $\mathbf{w}_{k+1} = \arg\min_{\mathbf{w}} z(\mathbf{w})$ will either reduce y or leave it unchanged.

Since the quadratic Taylor approximation of $\mathcal{C}(\mathbf{w})$ using $\mathbf{H}_{GN}$ in Equation A.1 is convex, it may serve as y. Chun & Fessler (2018) showed that because the diagonal elements of $\mathbf{H}_{GN}$ are all positive:

$$\mathbf{H}_{MM} = \operatorname{diag}\left(\left|\mathbf{H}_{GN}\right|\mathbf{1}\right) \succeq \mathbf{H}_{GN} \quad \text{(A.7)}$$

where $\left|\mathbf{H}_{GN}\right|$ is the matrix of the absolute values of $\mathbf{H}_{GN}$ and $\mathbf{1}$ is a column vector of ones. In simpler terms, $\mathbf{H}_{MM}$ is a matrix that is zero everywhere except along its main diagonal, and the value on the diagonal for any row is equal to the sum of the absolute values of the corresponding row in $\mathbf{H}_{GN}$. The $\succeq$ symbol means that $\mathbf{H}_{MM}$ is at least as positive definite as $\mathbf{H}_{GN}$. Therefore, substituting $\mathbf{H}_{MM}$ for $\mathbf{H}_{GN}$ in the quadratic Taylor approximation of $\mathcal{C}(\mathbf{w})$ majorises y, and may serve as z. In other words simply, replacing $\mathbf{H}_{GN}$ with $\mathbf{H}_{MM}$ in the LM algorithm and solving as usual satisfies the MM algorithm requirements.

The major advantage of using $\mathbf{H}_{MM}$ is that it is only non-zero along its main diagonal, and therefore requires far less memory to store than $\mathbf{H}_{GN}$. This is particularly important when the number of parameters in $\mathbf{w}$ is large, as it may not be possible to store $\mathbf{H}_{GN}$.
